# Supplementary material for: Utilizing Gold Nanoparticle Decoration for Enhanced UV Photodetection in CdS Thin Films Fabricated by Pulsed Laser Deposition: Exploiting Plasmon-Induced Effects
Source: Nanomaterials (Basel). 2024 Feb 24;14(5):416. doi: 10.3390/nano14050416 (PMC10933858; doi:10.3390/nano14050416)
Supplement: Supplementary file 1 [file nanomaterials-14-00416-s001.zip › nanomaterials-2762390-supplementary.pdf]

nanomaterials

Supplementary Information

Utilizing Gold Nanoparticle Decoration for Enhanced UV Photodetection in CdS Thin Films Fabricated by Pulsed Laser Deposition: Exploiting Plasmon-Induced Effects

Walid Belaid [1], Serap Yiğit Gezgin [1], Mohamed A. Basyooni-M. Kabatas [2,3,4,*], Yasin Ramazan Eker [4,5] and Hamdi Şükür Kiliç [1,6,7]

[1] Department of Physics, Faculty of Science, Selçuk University, Konya 42075, Turkey; walid.belaid@usmba.ac.ma (W.B.); serap.gezgin@selcuk.edu.tr (S.Y.G.); hamdisukurkilic@selcuk.edu.tr (H.Ş.K.)
[2] Dynamics of Micro and Nano Systems Group, Department of Precision and Microsystems Engineering, Delft University of Technology, Mekelweg 2, 2628 CD Delft, The Netherlands
[3] Solar Research Laboratory, Solar and Space Research Department, National Research Institute of Astronomy and Geophysics, Cairo 11421, Egypt
[4] Science and Technology Research and Application Center (BITAM), Necmettin Erbakan University, Konya 42090, Turkey; yeker@erbakan.edu.tr
[5] Department of Basic Sciences, Faculty of Engineering, Necmettin Erbakan University, Konya 42090, Turkey
[6] Directorate of High Technology Research and Application Center, University of Selçuk, Konya 42031, Turkey
[7] Directorate of Laser-Induced Proton Therapy Application and Research Center, University of Selçuk, Konya 42031, Turkey
* Correspondence: m.kabatas@tudelft.nl or m.a.basyooni@gmail.com

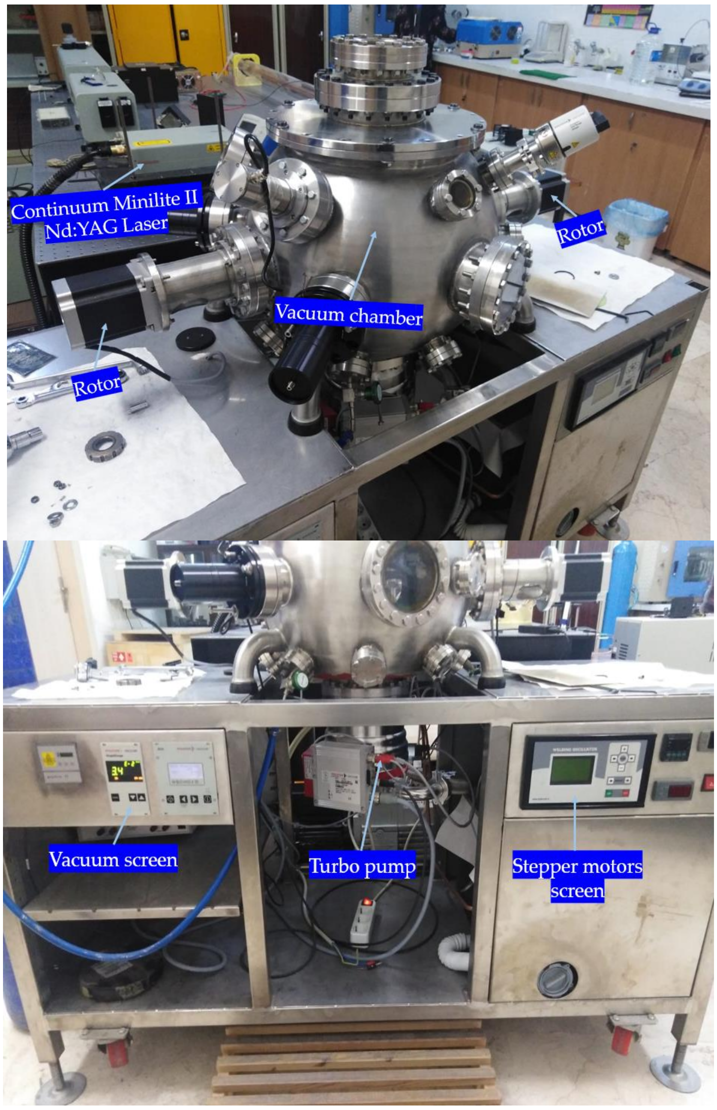

Figure S1. The used PLD system.

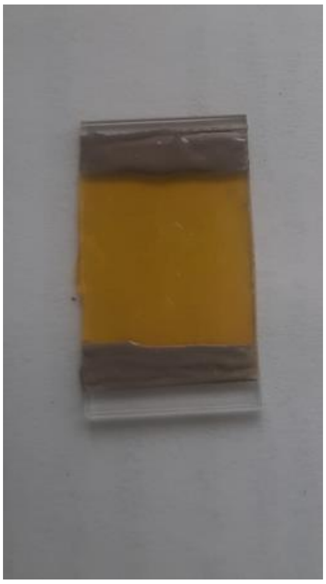

Figure S2. Photographic image of CdS thin film sample with Silver (Ag) Electrodes.

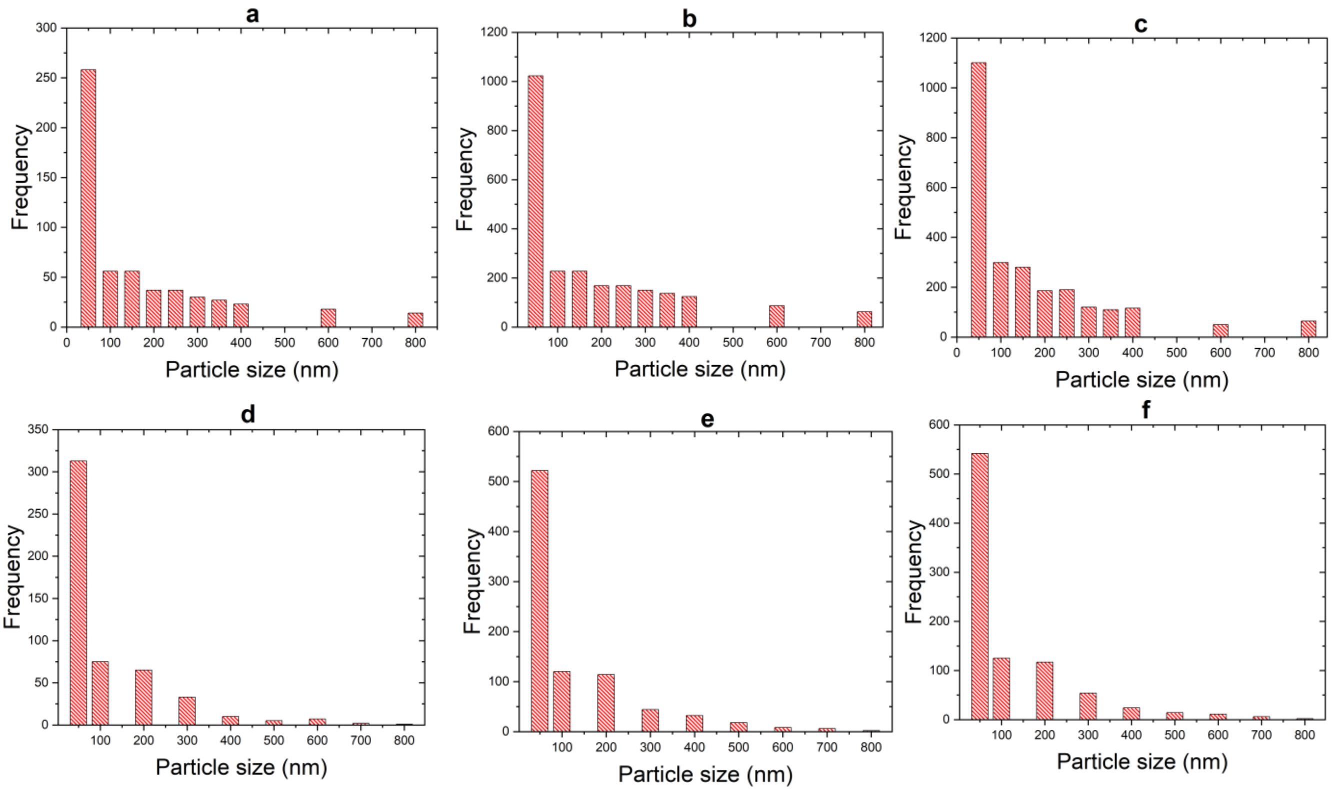

Figure S3. Frequency distribution of nanoparticle sizes for samples S1-S6, labeled (a-f) respectively. This histogram represents the uniformity in particle sizes as determined by ImageJ analysis providing information on the surface distribution of nanoparticles.

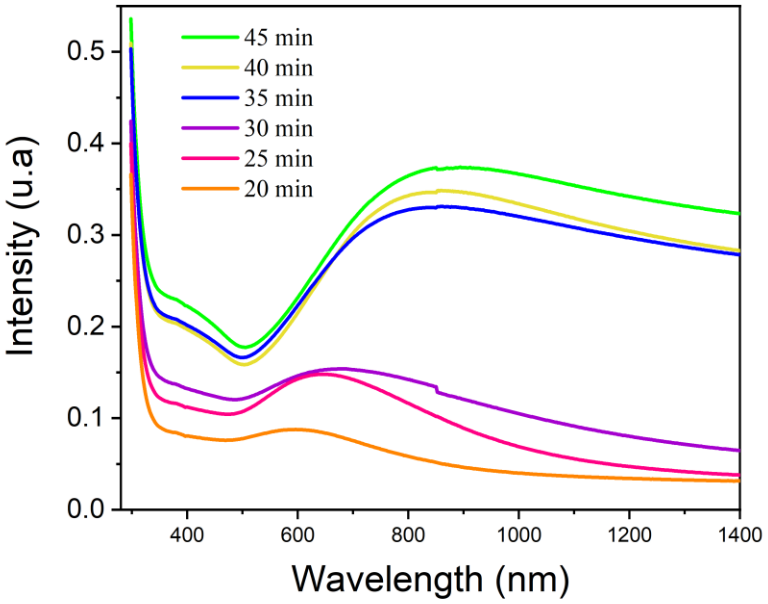

Figure S4. UV-Visible Absorption Spectra for Gold Nanoparticles deposited with an energy of 20 mJ at Room Temperature for different deposition time.
